# Solid-State Nanopore Sensors: Analyte Quantification by Event Frequency Analysis at High Voltages

**DOI:** 10.1021/acs.analchem.4c05037

**Published:** 2025-02-20

**Authors:** Julia Järlebark, Wei Liu, Amina Shaji, Jingjie Sha, Andreas Dahlin

**Affiliations:** † Department of Chemistry and Chemical Engineering, Chalmers University of Technology, 41296 Gothenburg, Sweden; ‡ Jiangsu Key Laboratory for Design and Manufacture of Micro-nano Biomedical Instruments & School of Mechanical Engineering, Southeast University, Nanjing 211189, China

## Abstract

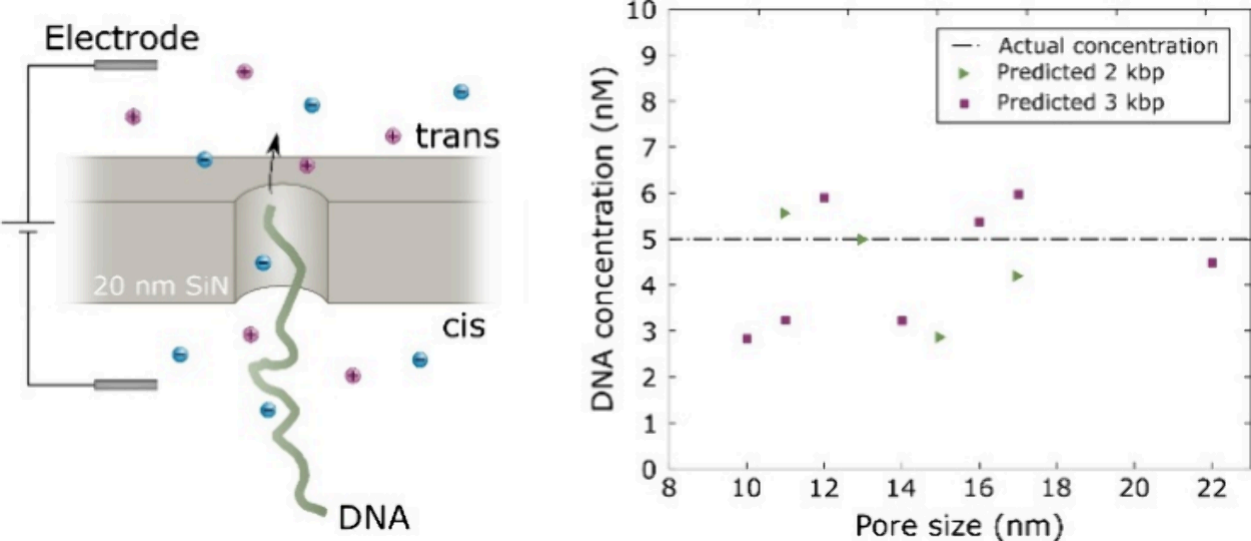

Solid state nanopores have emerged as an important electrical label-free single-molecule detection platform. While much effort has been spent on analyzing the current trace to determine size, shape and charge of the translocating species, a less studied aspect is the number of events and how this relates to analyte concentration. In this work we systematically investigate how the event frequency depends on voltage applied across the pore and show that this dependence can be utilized to determine target concentration. Importantly, this method does not require any calibration or any additional species added to the solution. Data analysis algorithms are introduced to accurately count events also for high voltages (up to 1 V). For double stranded DNA as model analyte, we find a linear relation between event frequency and voltage for pores 10 nm or more in diameter. For smaller pores, the majority of events are dockings rather than translocations and the linear relation is lost, in agreement with theory. Our model also predicts that the electrophoretic mobility of the species will influence event frequency, while diffusivity does not, which we confirm by using two different sizes of DNA. The analyte concentration determination is found to be remarkably accurate (10% error) when taking the average of multiple (∼4) experiments. If based on a single experiment, the predictive power is lower, but the method still provides a useful estimate (<30% error). This study should be useful as a guide when performing experiments at higher voltages and may serve as a method to extract analyte concentration in bioanalytical applications of nanopore sensors.

## Introduction

Nanopores have emerged as an important sensor technology for single biomolecule detection and analysis. With the exception of optical detection, the transduction mechanism is generally based on changes in the ionic conductance of a single pore when molecules are present inside,^1,2^ in analogy with the Coulter counter for detecting cells in microscale capillaries. The nanopores can be either solid state, biological, or hybrid variants.^3^ While biological nanopores are capable of sequencing DNA and recently also peptides,^4^ solid state nanopores are more robust and often suitable for other analytical approaches.^5^ Furthermore, even if sequencing is not feasible, detailed analysis of the current trace can reveal information about the size and shape of the species passing through the nanopore.^6^ It is also possible to detect specific interactions and perform affinity-based detection using either receptors immobilized inside the pore^7^ or with both species free in solution.^8^ In this context, data analysis algorithms that extract and analyze translocation events from the current trace are critical.^9,10^

While the methodology to date has focused mostly on analysis of signal magnitude and dwell time,^11^ a fundamental parameter that is more rarely discussed is the event frequency, i.e., how often a molecule will pass through the pore. Accurately determining the event frequency is clearly a necessity (though not a guarantee) for obtaining the concentration of the translocating species,^10^ which is arguably the most central parameter in bioanalytical applications. Obtaining the frequency of translocation events is also the basis for analyzing transport selectivity.^5,12,13^ Furthermore, analysis of how the event frequency depends on the voltage applied across the pore membrane provides information about what limits the transport rate: diffusion to the pore or the translocation event itself.^14^ This becomes especially important when nanopores have been chemically modified for the purpose of achieving a selective barrier. As an example, when studying spontaneous protein translocation through nuclear pore mimics,^15^ it is essential to verify that the applied voltage remains noninvasive and does not influence the event frequency.^13^ Similarly, analyte binding to receptors inside the pore may well be influenced by the extremely high local field (∼10^7^ V/m). Investigating this effect requires altering the voltage, but such tests are only rarely performed.^15,16^ One reason is the known issue with poorer baseline stability as well as increased short-term noise at higher voltages (>200 mV), which is often observable in the data trace.^3^

In this work, we present a new method for obtaining the analyte concentration based on accurate determination of the event frequency over a broad voltage range. The analysis does not require any kind of calibration and can be applied directly on a data set, given that translocations were measured at different voltages. We overcome common issues when measuring at higher voltages by introducing data analysis algorithms to remove baseline instabilities and accurately count events. To the best of our knowledge, this is the first study that shows how the relation between event frequency and voltage directly can provide the analyte concentration, without any additional species introduced to the sample. The methodology and the algorithms (a Matlab implementation is appended) should be highly useful for any kind of nanopore sensing application where the event frequency is an important parameter or where the analyte concentration is of interest to determine.

## Results and Discussion

### Theory of Event Frequency

We start by giving the theoretical background of the event frequency. Our treatment here is similar to previous work.^10,14,17−19^ Further details on the derivation are given in the Supporting Information. The electric field generated by the applied DC voltage Δ*U* is generally focused to the pore, but will also be present outside. The potential will vary radially as
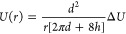
1Here *d* is the pore diameter, while *h* is the membrane thickness and *r* = 0 represents the middle of the circular pore opening (Figure S1). The fact that there are two terms in the denominator is due to access resistance.^20^ In the bulk reservoirs, free diffusion will dominate over electrophoretic motion, but close to the pore molecules will be “captured” by the strong local electric field. The critical distance from the pore where this occurs is
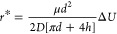
2Here, μ is the electrophoretic mobility and *D* is the diffusion constant. The highest event frequency is obtained when diffusion to the half-spherical capture zone is the rate limiting step, i.e., when molecules translocate as soon as *r* < r*. It should be noted that r* is expected to be on the order of a few microns.^21^ For such small values, the capture zone is point-like compared to the larger reservoirs, which means that the incident flux becomes constant very fast and we get the maximal (diffusion-controlled) event frequency *f*_0_ as
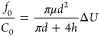
3Here *C*_0_ is the bulk concentration of the translocating species. Interestingly, despite representing the solution to a diffusion-controlled mass transport problem, eq 3 predicts that *f*_0_ does not depend on the diffusion constant of the species, while the electrophoretic mobility does come into play. This means that if the model is correct, for DNA,^22^ the number of base pairs should not influence event frequency. In addition, *f*_0_ is predicted to be proportional to Δ*U*. A few experimental studies have confirmed this relation.^10,17,21^ It can also be noted that if the linear behavior is observed, it should be possible to determine *C*_0_ given that the pore dimensions and μ are known. However, this approach to determine analyte concentration seems not tested in any study to date.

When the pore represents a significant barrier for translocation, molecules will accumulate instead of becoming depleted at the pore opening. When the translocation event itself is fully rate-limiting, an equilibrium concentration distribution will be established and the increased probability of finding a molecule at a certain distance from the pore should be given by a Boltzmann factor:
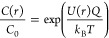
4Here *Q* is the net charge of the molecule. Since *U*(r) is proportional to Δ*U* (eq 1), and since the “attempt frequency” to enter the pore should be proportional to the local concentration at the pore, an exponential dependence of event frequency on Δ*U* emerges. As discussed by Wanunu et al.^14^ this is the case even without considering the free energy barrier associated with entering the pore. In other words, the size of the molecule and its required conformational changes may well explain why the pore represents a barrier, but an exponential dependence of event frequency with voltage will follow regardless of how (if) the voltage influences the probability of obtaining the right conformation. Experimentally, the exponential dependence has been observed for DNA threading through pores a few nm in diameter^10,14^ and becomes particularly clear for biological nanopores.^23^

### Data Analysis

**Figure 1:**
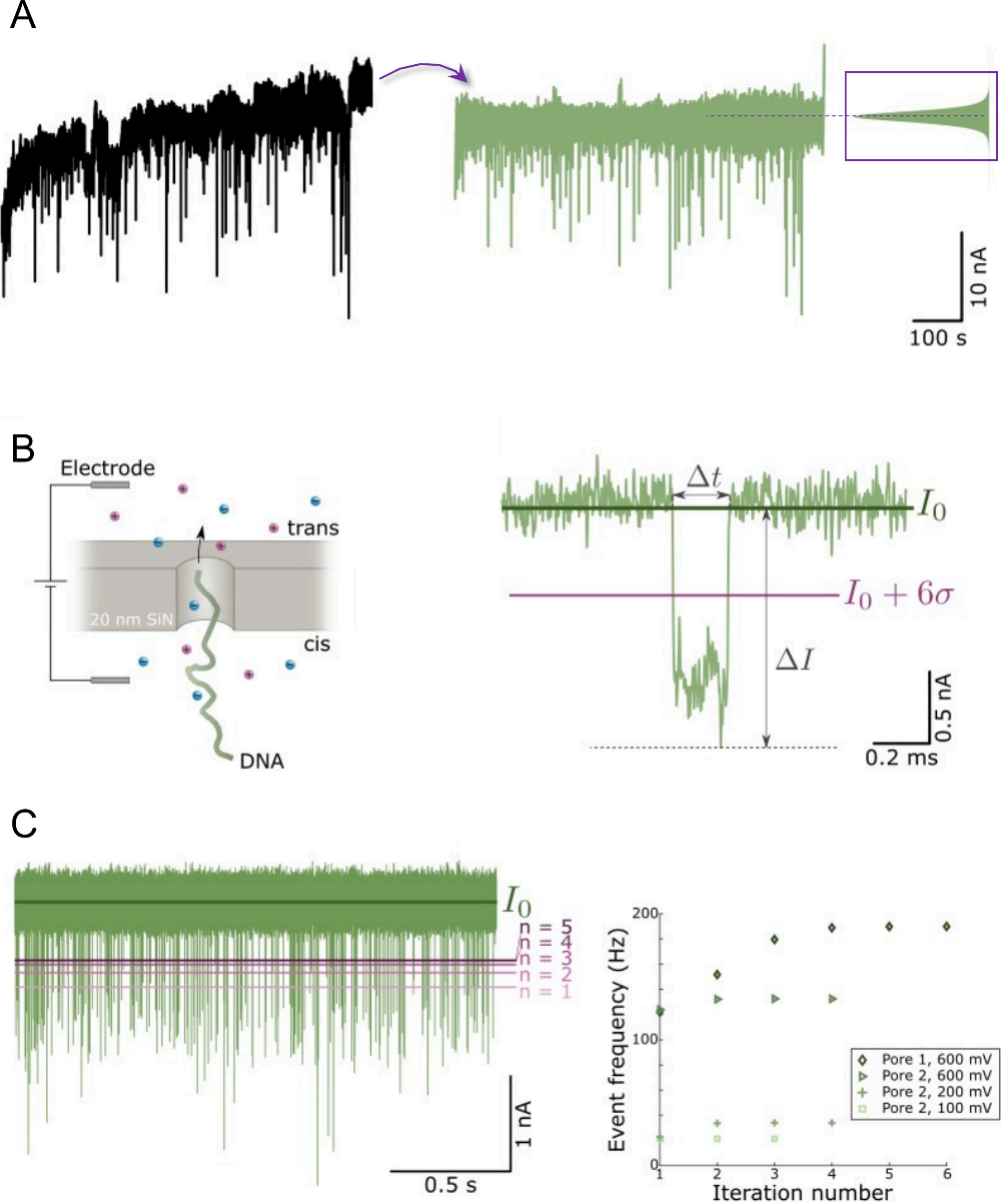
Data analysis for accurate event frequency determination at high voltages. (A) Example of baseline correction by Fourier transform and high-pass filtering. The resulting baseline (measured at 0.9 V) has a Gaussian distribution. (B) Schematic of DNA detection and example of a translocation event, showing definitions of threshold value, signal magnitude Δ*I* and dwell time Δ*t*. (C) Iterative event counting by generation of new threshold values. At high voltages (e.g., 600 mV) several iterations are sometimes needed for converging to the accurate value of *f*.

To clearly resolve the relation between event frequency *f* and Δ*U*, the voltage interval should ideally be as wide as possible. However, a common issue when measuring at higher voltages is baseline instability, which makes it difficult to identify events correctly in the current trace. The obvious solution for many experiments is to simply stay at low voltages (∼0.1 V), but here our aim is to measure event frequencies at high voltages to thoroughly investigate the validity of eq 3 and whether it can be used to obtain the analyte concentration. To improve baseline stability we performed fast Fourier transforms of the current time trace into its frequency components. The lowest frequency components, corresponding to baseline fluctuations, were then eliminated from the data. A typical cutoff value which gives the desired result is 100 Hz, but this can be easily tuned depending on the application. The data was then inverse transformed back to the time domain to regain the current trace, with a stable baseline. An example of the effect of this baseline-correction algorithm is shown in Figure 1A. We emphasize that accurate counting of events was not possible without this signal treatment.

After high-pass filtering, events in the current trace could be identified using a simple threshold detection algorithm. The threshold for an event was set as a fixed number of standard deviations (σ) from the mean of the baseline corrected current trace (Figure 1B), typically 6σ. The choice of threshold obviously impacts the number of detected events, where a balance is sought to avoid missing translocation events or introducing false positives due to a too low threshold. To verify that no false events were detected, threshold values were tested on the baseline recorded before DNA was introduced. Furthermore, the end of an event was registered at the first current value to be higher than the mean, thereby defining the dwell time Δ*t* (Figure 1B). The amplitude Δ*I* of an event was obtained as the difference between the mean current and the value deviating the most from the baseline during the event.

While our definition of events in the current trace as well as their Δ*I* and Δ*t* values are conventional,^11^ problems emerge for higher event frequencies, which naturally occur at higher voltages. It is no longer accurate to calculate σ based on the entire time trace if a molecule is present in the pore a significant fraction of the time, simply because this situation does not represent the baseline. To circumvent this issue, we used an iterative approach, partly inspired by previous work.^9^ First, all events initially detected by the algorithm described above were eliminated by setting all current values within the dwell time of an event equal to the mean current value of the rest of the trace. For the new current trace that resulted, a new mean and standard deviation were calculated, and the threshold detection was performed again with these new parameters, but on the original current signal. Additional events were then detected because σ was lower (and more accurately representing the baseline). This event detection procedure was then iterated until the number of detected events no longer increased. Convergence was usually observed already after 2–3 iterations, but the influence on event frequency was sometimes very large (Figure 1C), illustrating the importance of this treatment to determine high *f* values accurately.

As a final point on the data analysis, it should be noted that nanopores are known to sometimes enter a blocked state where no translocations occur for some time. (For instance, this may be due to a bubble formed at the pore.) If the pore is actually blocked a significant part of the time, the event frequency obviously becomes underestimated. One cumbersome solution is to manually exclude such regions from the time trace. However, it is better to check the intermittent time in between events,^10^ which should follow a Poissonian probability distribution with a characteristic decay time equal to the inverse of the event frequency. Even if the pore is blocked for a long time, this only affects a single value in the distribution of intermittent times, so the influence on the event frequency becomes negligible. For our experiments, we verified that the intermittent time analysis approach provided the same *f* values as conventional event couning (Figure S2).

As Supporting Information for this paper we provide a Matlab implementation of all the data analysis algorithms described.

### High Voltage Measurements

**Figure 2:**
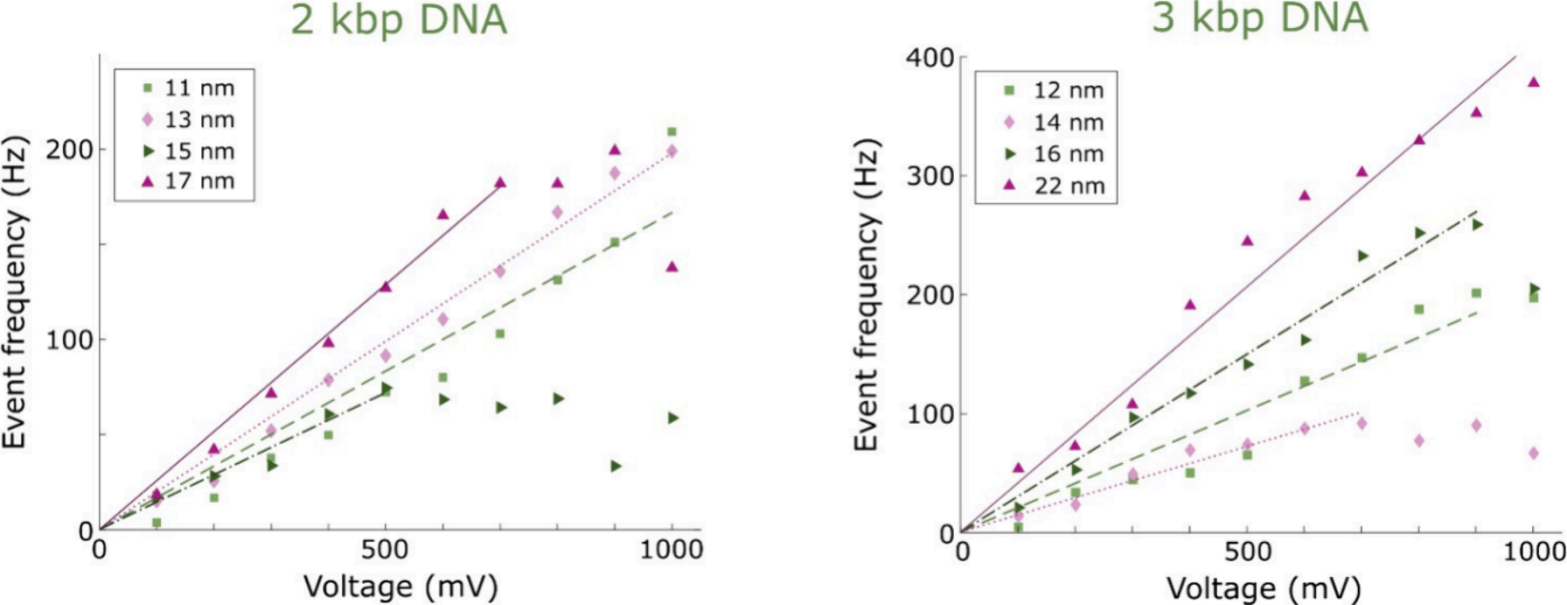
Example data of event frequencies determined at voltages up to 1 V using the data analysis approach for accurate event counting. To maximize accuracy for each data point, all events were included when calculating *f* for a given voltage (hence no error bars). The lines show linear fits (forced through origin, i.e., without a constant term) to the data up until the first *f* value that no longer increases with Δ*U*. The points that deviate from the linear trend are due to limited instrument bandwidth.

Nanopores in silicon nitride membranes were prepared by controlled dielectric breakdown^24^ (CDB) and double stranded DNA with sizes of 2 or 3 kbp was used for all translocation experiments. Figure 2 shows event frequencies at different voltages and for different nanopore diameters. All *d* values were determined based on the conductance (Figure S3). A linear relation between *f* and Δ*U* was observed all the way up to 1 V in most cases for pores 10 nm or more in diameter when performing the data analysis as described above. We also verified that our signal magnitudes were in agreement with the expected ones for DNA translocation^20^ (Figure S4). Deviations from a linear behavior, i.e., curves more similar to an exponential dependence, as suggested by eq 4, were observed for pores below 10 nm in size. This also coincided with emergence of “docking” or “collision” events^25^ rather than translocations, as concluded based on strongly deviating values for Δ*I* and Δ*t* (Figure S5). The explanation for this behavior is simply that double stranded DNA cannot easily enter very small pores. Since the model for *f*_0_ assumes that translocation occurs as soon as molecules are close to the pore, i.e., *C*(*r* < r*) = 0, such events indeed indicate that eq 3 is no longer applicable because the pore has become a significant barrier. Regarding the observed cutoff value of *d* = 10 nm, it should be noted that pores prepared by CDB are not necessarily perfectly circular in their cross-section^26^ and the interior walls may not be fully vertical. Hence, all values for *d* represent an effective diameter equivalent to a cylindrical pore with respect to conductance. Nevertheless, we hypothesize that in our electrolyte (1 M KCl) the double stranded DNA is simply not compacted enough for easy translocation when *d* < 10 nm.

**Figure 3:**
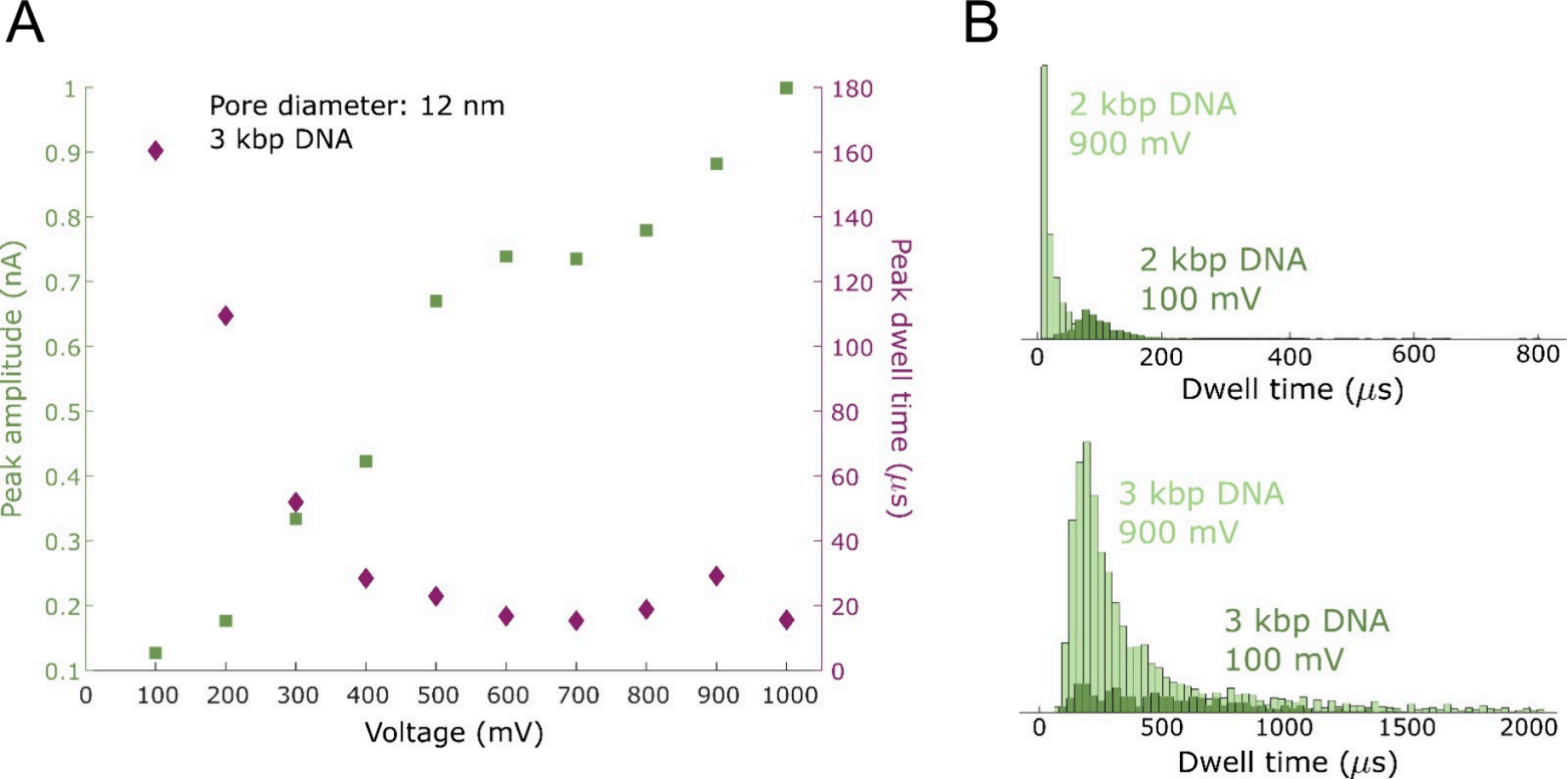
(A) Typical plot of signal magnitude and dwell time vs voltage. The amplitude increases linearly as expected. The dwell time decreases due to the enhanced electrophoretic force and appears to saturate after 500 mV due to limited sampling rate. (B) Examples of dwell time distributions for different DNA lengths and voltages.

We also analyzed how signal amplitude Δ*I* and dwell time Δ*t* were affected by the high voltages (Figure 3A). While Δ*I* was linear with voltage as expected, i.e., the conductance change remained the same, Δ*t* was gradually reduced as a result of the stronger electrophoretic force acting on DNA. In fact, especially for the shorter DNA strand (2 kbp), the dwell time became comparable to the sampling rate of the measurement, as shown by the statistical distributions at higher voltages (Figure 3B). This illustrates a potential pitfall because events will be missed, leading to an underestimated *f*. Indeed, for all the data points at higher voltages that deviated downward in Figure 2, we could confirm from the dwell time distributions that events were missed (and therefore the corresponding *f* values were not included in the analysis below). As expected, this occurred primarily for the shorter 2 kbp DNA as it translocates faster. A possible solution is to increase the sampling rate, but this leads to higher short-term noise and a risk of missing events due to 6σ becoming comparable to Δ*I*, at least for our current experimental setup. Hence the points in Figure 2 that deviate from the linear trend are simply a result of instrumental and sample limitations. The best way to verify that no significant number of events are missed is to see that the asymmetric dwell time distribution^27^ approaches zero before the limit defined by the sampling rate, i.e., that a peak is visible, and that Δ*I* > 6σ. We note that one can also perform measurements in high concentrations of LiCl to increase dwell time.^28^ Nevertheless, our current results are sufficient for evaluating the method for concentration determination.

### Determination of Analyte Concentration

Finally, we show that the plots of *f* vs Δ*U* can be used to determine analyte concentration. Given that this relation is linear, the slope of the line should be given by eq 3. For the electrophoretic mobility of DNA in 1 M salt, we used the value μ = 1.5 × 10^–4^ cm^2^ V^–1^ s^–1^ from Stellwagen and Stellwagen.^29^ Note that this value is fairly generic since μ does not depend on DNA length, while *D* does. We note that in principle, it should be possible to obtain μ from the dwell time distributions (Figure 3B) using appropriate drift-diffusion models. However, this has been tested by Li and Talaga,^27^ who concluded that the resulting μ values were around an order of magnitude too small. Hence, we propose to use electrophoretic mobility values that are independently determined, at least until models for extracting μ from dwell time data have improved. Note that our method should work analogously for proteins but the value of μ needs to be changed accordingly.

**Figure 4:**
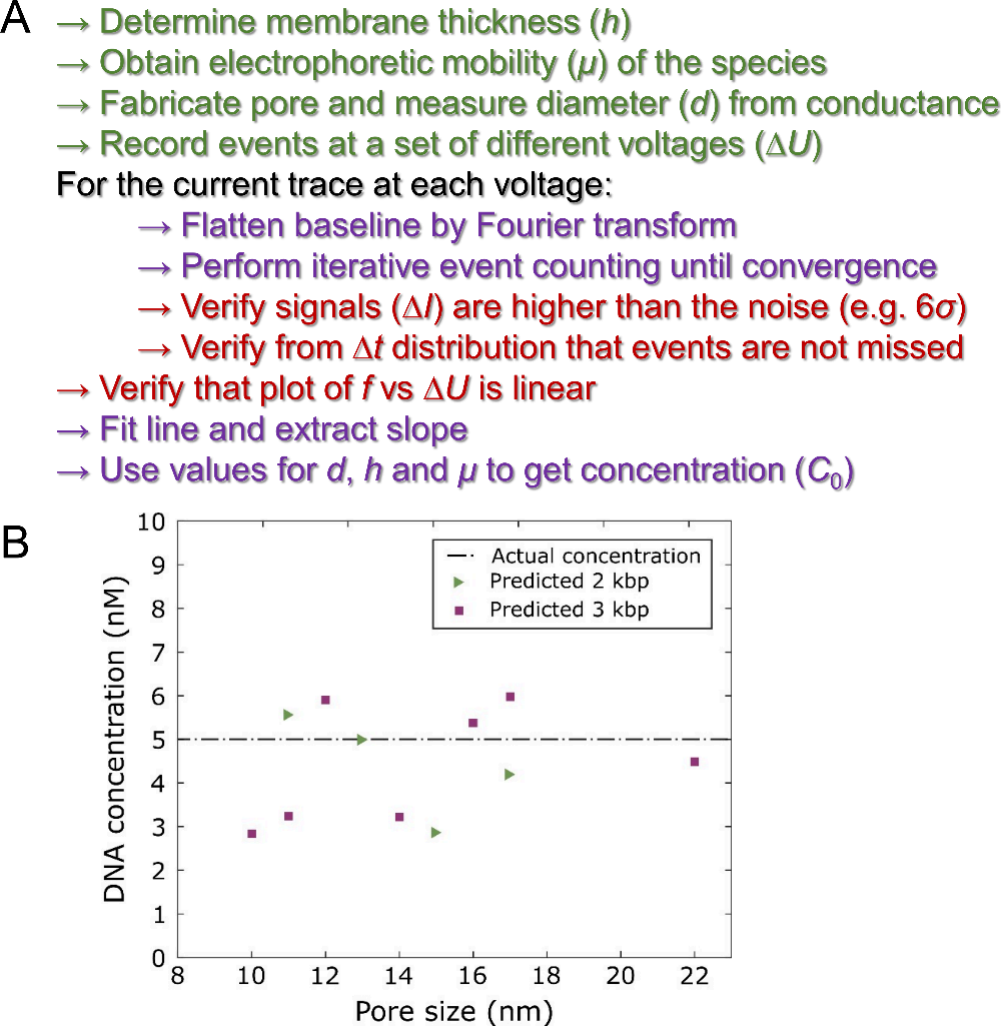
(A) Description of the method for analyte concentration determination. Green: experimental work. Purple: data analysis steps. Red: sanity checks. (B) Plot of predicted analyte concentration for different pore diameters and two different DNA lengths. Each data point represents one experiment where the voltage was varied. The actual concentration was 5 nM in all cases.

In the analysis, we excluded all values for *f* where it was obvious from the dwell time distribution (Figure 3B) that not all events could be properly counted due to the limited sampling rate. (This was eventually confirmed by performing a few additional experiments with a higher bandwidth instrument.) To be precise, the first *f* value that no longer increased with Δ*U* and all values thereafter (for higher Δ*U*) were removed when performing the linear fitting. Besides *d*, a value for *h* is also needed to utilize eq 3. This was obtained by spectroscopic ellipsometry of the SiN_*x*_ coated Si wafer after KOH etching (for membranes made in-house) or from the manufacturer (for purchased membranes). The point-by-point methodology to obtain the analyte concentration is summarized in Figure 4A. Note that there is no constant term in the linear fit, i.e., it is forced to go through origin.

Figure 4B shows an example of predicted (*C*_0_) vs actual analyte concentration for different nanopore diameters. It can first be seen that there is no trend for the smaller 2 kbp vs the larger 3 kbp DNA, confirming that only the electrophoretic mobility is important, not the diffusivity. It can also be noted that there is no trend of overestimation or underestimation for different pore diameters. The average predicted concentrations for 2 and 3 kbp were both 4.4 nM and the actual concentration was 5.0 nM in both cases. The standard error was ±1.2 nM for 2 kbp (*n* = 4) and ±1.3 nM for 3 kbp (*n* = 6). This means that a single plot of *f* vs Δ*U* has limited predictive power, but still gives a fair estimate. The main source of variation is likely that the model is based on pores that are circular, while fabrication by CDB does not necessarily provide this shape. Also, any small increase in diameter during the measurements gives a significant effect on the concentration determination.

## Conclusions

We have addressed the challenge of analyte concentration determination with solid state nanopore sensors and accurate determination of event frequency at high voltages. Furthermore, we have investigated the relation between voltage and event frequency in detail. The signal processing algorithms presented here enable accurate detection and quantification of translocation events up to 1 V or even more depending on the dwell time and the sampling rate of the instrument. A theoretical treatment shows that for a barrier-free pore (diffusion-limited transport), the relation between voltage and event frequency is linear and this is confirmed experimentally for pores larger than 10 nm. By using two sizes of DNA we confirmed that diffusivity plays no role: only the electrophoretic mobility of the species is needed. The derived expression in eq 3 makes it possible to determine analyte concentration accurately, at least when taking the average of several measurements. We emphasize that proper data analysis is important to accurately count events at higher voltages to verify the linear relation between *f* and Δ*U* and to determine the slope.

We believe these results can be useful for the research field of solid state nanopore sensors. For instance, accurate counting of translocation events is indeed important in recent application such as molecular delivery to single cells.^30^ The concentration determination is particularly attractive since there is no need for a calibration. Importantly, the method in Figure 4A still works even if the sample contains multiple species as long as the relevant events, i.e., those caused by the analyte molecule, can be sorted out based on their ion current trace characteristics. For instance, it has already been proven feasible to detect targets bound to translocating aptamers through the extra resistive pulses.^16^

### Experimental Section

Silicon nitride membranes were prepared as described previously^31^ or purchased from Norcada. CDB and pore conditioning (size tuning) was performed as described previously^32^ using a SPARK-E2 from Northern Nanopore Instruments. Spectroscopic ellipsometry to determine SiN_*x*_ membrane thickness was performed using a J.A. Woollam RC2. Pores that were quickly increasing in size during an experiment (as determined from conductance) or with unusually high short-term noise were excluded. All translocation data were collected in a 1 M KCl solution with 10 mM Tris and 1 mM EDTA at pH 8 using an Axopatch 200B or (in a few cases) an Elements Nanopore Reader 10 MHz. Data was typically collected for 1–2 min at each voltage. (Longer measuring improves accuracy in *f* values but increases the risk of significant pore growth.) The bandwidth was set to 10 kHz for lower voltages and 100 kHz for higher voltages when using the Axopatch. The appended software reads data files in ABF (Axon Binary File).

## Supporting Information Available

Theory of event frequency, intermittent time analysis, determination of pore diameter, derivation of signal magnitude, and events in very small pores (PDF)
Matlab codes Event_analyzer.m and abfload.m (ZIP)
