# Supplementary material for: Solid-State Nanopore Sensors: Analyte Quantification by Event Frequency Analysis at High Voltages
Source: Anal Chem. 2025 Feb 20;97(8):4359–64. doi: 10.1021/acs.analchem.4c05037 (PMC11883732; doi:10.1021/acs.analchem.4c05037)
Supplement: Supplementary file 1 — ac4c05037_si_001.pdf [file ac4c05037_si_001.pdf]

# Solid-State Nanopore Sensors: Analyte Quantification by Event Frequency Analysis at High Voltages

*Julia Järlebark,[1] Wei Liu,[2] Amina Shaji,[1] Jingjie Sha[2] and Andreas Dahlin.[1]*

1 Department of Chemistry and Chemical Engineering, Chalmers University of Technology, 41296 Gothenburg, Sweden.

2 Jiangsu Key Laboratory for Design and Manufacture of Micro-nano Biomedical Instruments & School of Mechanical Engineering, Southeast University, Nanjing 211189, China.

**Table of Contents**

Theory of event frequency, including Figure S1 (pages 2-4).

Event frequency by intermittent time analysis, including Figure S2 (page 5).

Determination of pore diameter, including Figure S3 (page 6).

Derivation of signal magnitude, including Figure S4 (page 7).

Events in very small pores, including Figure S5 (page 8).

**Detailed theory of event frequency**

For completeness, we derive the expected dependence of event frequency on voltage from scratch. As explained in the main text, the derivation is partly overlapping with previous literature.[1]

Considering the circuit as a cylindrical pore with diameter *d* in a membrane with thickness *h*, the total resistance is that of the pore ($R_{\text{pore}}$) plus two access resistances ($R_{\text{access}}$) that should be equal for a symmetric pore. These are given by:[2]

$$R_{\text{pore}} = \left[\frac{g\pi d^2}{4h}\right]^{-1}$$

$$R_{\text{access}} = [2dg]^{-1}$$

Here *g* is the conductivity of the medium (the electrolyte). If one side is grounded, the potential at infinite distance is $U = 0$, while on the other side the potential approaches that applied in the measurement ($\Delta U$). If the potential at the pore opening is $U_{\text{pore}}$, the potential drop over the pore is $\Delta U - 2U_{\text{pore}}$ by symmetry. If the current is *I* this gives, first of all, the well-known relation used to determine *d* from the measured conductance:

$$\Delta U = \frac{I}{g}\left[\frac{1}{d} + \frac{4h}{\pi d^2}\right]$$

Next, we consider the potential drop in the reservoirs outside the pore. In this context the pore is small and can be approximated as a point to which the current is focused in a half-sphere with radial symmetry. The potential drop over a shell with thickness d*r* is then:

$$\mathrm{d}U = \frac{I}{2\pi g r^2}\mathrm{d}r$$

We integrate this expression and set the potential to zero at infinite distance:

$$U(r) = \frac{I}{2\pi g r}$$

Using the relation between $\Delta U$ and *I* we can write:

$$U(r) = \frac{\Delta U}{r\left[\frac{2\pi}{d} + \frac{8h}{d^2}\right]}$$

---

[1] Wanunu, M.; Morrison, W.; Rabin, Y.; Grosberg, A. Y.; Meller, A., Electrostatic focusing of unlabelled DNA into nanoscale pores using a salt gradient. *Nature Nanotechnology* **2010,** *5*, 160-165.

[2] Kowalczyk, S. W.; Grosberg, A. Y.; Rabin, Y.; Dekker, C., Modeling the conductance and DNA blockade of solid-state nanopores. *Nanotechnology* **2011**, 22, 315101.

The field is then:

$$E(r) = \frac{\partial U}{\partial r} = -\frac{\Delta U}{\left[\frac{2\pi}{d} + \frac{8h}{d^2}\right] r^2} = -\frac{U(r)}{r}$$

Next, we find the characteristic distance $r^*$ from the pore opening where electrophoretic motion will dominate over diffusion for any $r < r^*$ (Figure S1). We compare the diffusive speed $r$ / $[r^2/D]$ with the electrophoretic speed $\mu|E|$, where $D$ is the diffusivity ($m^2s^{-1}$) and $\mu$ is the electrophoretic mobility ($m^2s^{-1}V^{-1}$). This gives $\mu U(r_c) = D$ which in turn gives the "capture radius" $r^*$ as described in the main text.

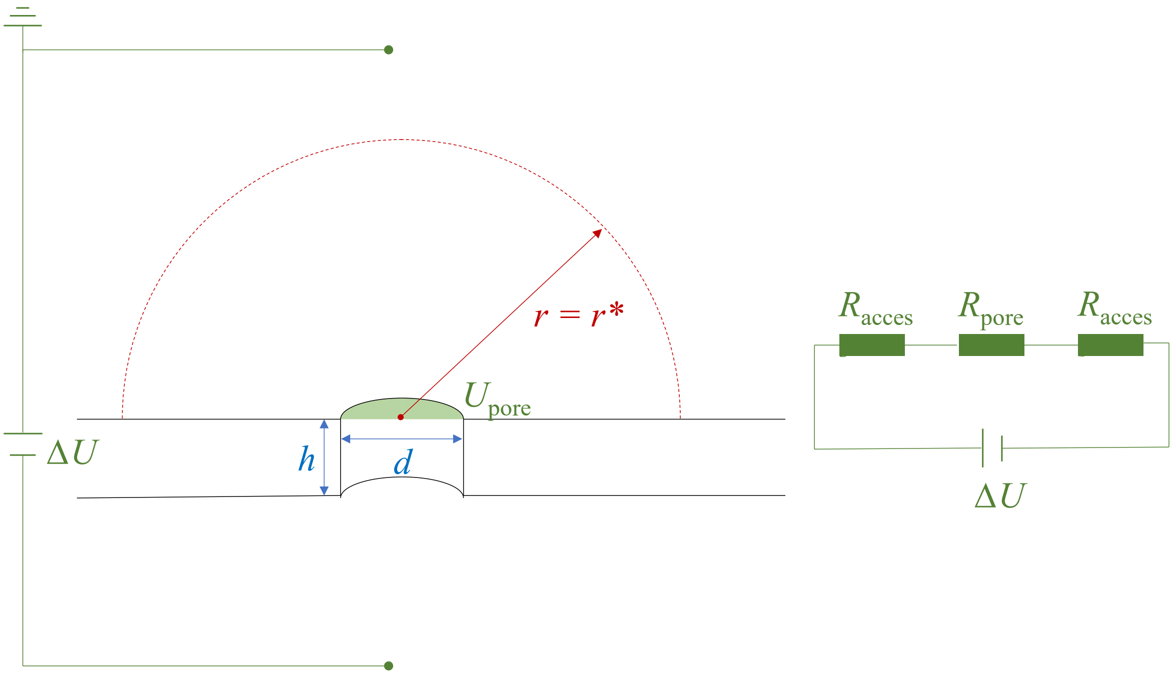


**Figure S1** Model of nanopore conductance with two access resistances in series with the cylindrical pore. The capture radius $r^*$ is the distance from the center of the pore opening at which molecules transition from free diffusion to electrophoretic drift motion through the pore.

Finally, to get the event frequency for a pore that has no barrier, we consider the free diffusive transport into the "capture zone" formed by the half-sphere with radius $r^*$. The solution is well-

known from electrochemistry with microelectrodes. It is the solution of Fick's law in spherical coordinates with a perfect "absorber" at a certain radius:[3]

$$J = DC_0 \left[\frac{1}{r^*} + \frac{1}{\sqrt{\pi D t}}\right]$$

Here $J$ is the diffusive flux and $C_0$ is the bulk concentration. Interestingly, the time dependence can be neglected when the electrode is sufficiently small, i.e. already after very short times only the $1/r^*$ term becomes significant, as can be tested by inserting typical values for $D$ (or by electrochemical experiments). This is because the depletion zone, where the concentration is lower than in the liquid bulk, disappears in the limit of a point electrode and spherical symmetry. Thus, we can remove the time dependent term and get the constant event frequency for a barrier-free pore by multiplying the flux with the area of the half sphere:

$$f_0 = \frac{4\pi r^{*2}}{2} \times \frac{DC_0}{r^*} = 2\pi D C_0 r^*$$

This gives the expression in the main text.

---

[3] Muthukumar, M., Theory of capture rate in polymer translocation. *The Journal of Chemical Physics* **2010,** *132*, 195101.

**Intermittent time analysis**

Assuming that translocation events occur independently and follow Poissonian statistics, the probability distribution of intermittent times $t_{int}$ follows a decay given by $\exp(-t_{int}/\tau)$. After fitting the exponential decay function the event frequency is obtained as $f = 1/\tau$. Figure S2 shows that this analysis yields the same result and an example of a distribution of $t_{int}$.

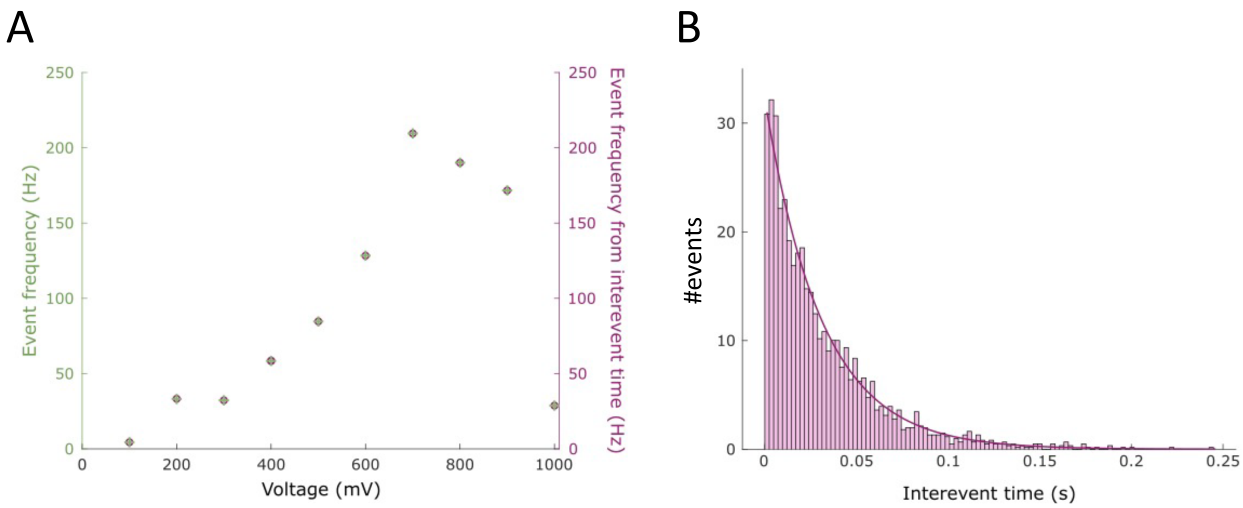


**Figure S2** (A) Event frequency determination by the time elapsed in between events gives the same result (data points differ by <1%). (B) Example of a distribution of intermittent times ($\Delta U$ = 300 mV, $d$ = 12 nm) and the exponential fit to determine $\tau$.

**Diameter determination**

The pore diameter is given by the pore conductance $G$ as:

$$d = \frac{G}{2g} + \sqrt{\frac{G}{g}\left[\frac{G}{4g} + \frac{4h}{\pi}\right]}$$

This model was used to determine $d$ based on IV curves after pore formation and conditioning, as exemplified in Figure S3.

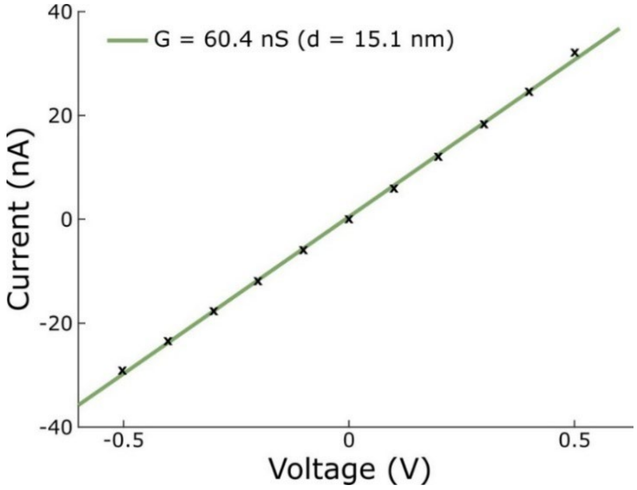


**Figure S3** Example of current at different voltages for a pore formed by CDB. The conductance $G$ is determined from the linear fit and used to calculate the pore diameter.

**Theoretical signal magnitude**

When a DNA strand is passing through the pore the cross-section area in the cylindrical volume is reduced by that corresponding to the cross-section area $A$ of the molecule, with $A$ = 380 Å$^2$ for dsDNA.[4] The conductance during a translocation is then:

$$G_{\mathrm{DNA}} = g\left[\frac{4h}{\pi d^2 - 4A} + \frac{1}{d}\right]^{-1}$$

This expression was used to estimate the expected signal amplitude. Figure S4 compares the measured amplitudes and calculated ones, i.e. $G - G_{\mathrm{DNA}}$, confirming the events are translocations.

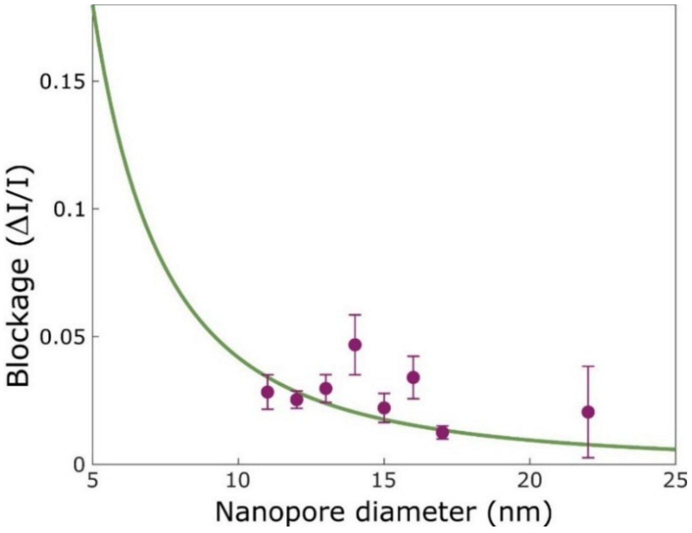


**Figure S4** Example of measured (data points) and calculated (solid line) relative current changes for different pore diameters. The calculation is based on a double stranded DNA molecule as it translocated the pore in a linear configuration.

[4] Kowalczyk, S. W.; Grosberg, A. Y.; Rabin, Y.; Dekker, C., Modeling the conductance and DNA blockade of solid-state nanopores. *Nanotechnology* **2011**, 22, 315101.

**Events in pores smaller than 10 nm**

An example of event distributions for a 6 nm pore is shown in Figure S5. Almost all events show signal magnitudes and dwell times are very different from those of the translocation events in larger pores. This is illustrated by the average amplitude and dwell time for a 12 nm pore (at the same voltage). When fitting *f* to Δ*U*, an exponential dependence emerges. This is because the probability that a DNA molecule “docks” in the pore is proportional to the local concentration at the opening and when the pore is so small that it constitutes a barrier, molecules will accumulate at the opening due to the electric field.

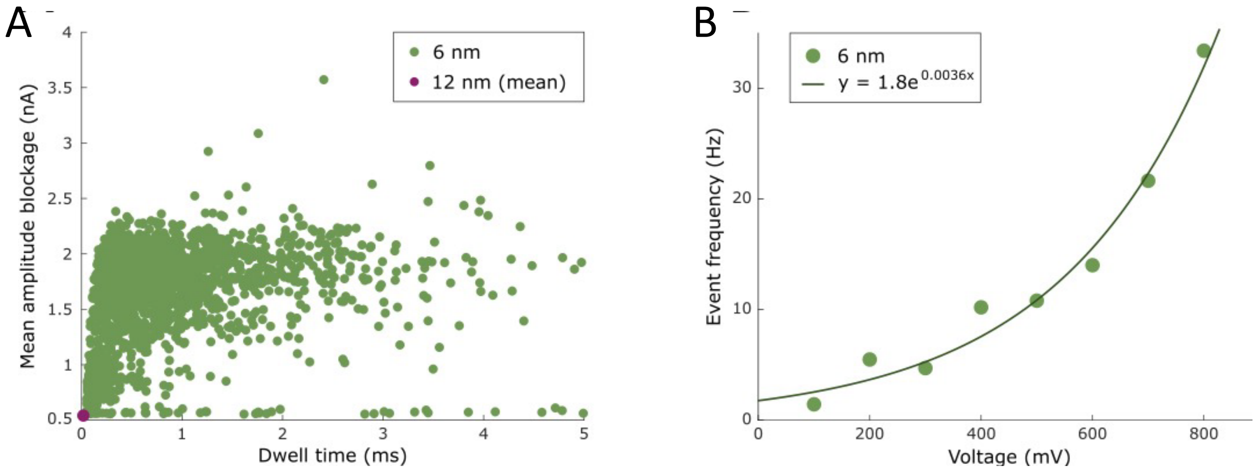


**Figure S5** (A) Scatter plot of signal amplitude and dwell time for a very small pore (*d* = 6 nm). For comparison, the average amplitude and dwell time for a larger pore (*d* = 12 nm) is also shown. Data obtained at Δ*U* = 400 mV. (B) Event frequency vs voltage for the 6 nm pore shows a curve that fits better to an exponential function than a line.
